# Split-screen distraction: the role of extraneous visual demands in learning from video

**DOI:** 10.1186/s41235-026-00720-2

**Published:** 2026-03-25

**Authors:** Brendan A. Schuetze

**Affiliations:** Department of Educational Psychology, University of Utah, 1721 Campus Center Drive RM 3220, Salt Lake City, UT 84112 USA

**Keywords:** Sludge content, Split-screen videos, Split attention, Multimedia, Multitasking

## Abstract

A genre of online videos known as “sludge content” has recently surged in popularity. These videos typically present two clips simultaneously, with one primary and one muted secondary video, creating an intentionally overstimulating viewing experience. Given the reliance on overstimulation and the inherent multitasking demands, these videos raise questions relevant to theories of multimedia learning and cognitive load. Inspired by this content format, a series of within-person studies was conducted to test whether simultaneous split-screen videos lead to changes in comprehension and memory. Two preregistered within-person studies (*N*_Study 1_ = 75, *N*_Study 2_ = 100) were employed to examine whether simultaneous split-screen presentations impaired comprehension or memory. Contrary to predictions, no strong evidence that simultaneous video presentation affected memory was found. Limited self-reported differences in interest with higher interest reported for non-split-screen videos were found in Study 2, but no differences in attention difficulty or cognitive load. These findings suggest that viewers may adapt to extraneous split-screen visual input more effectively than commonly assumed.

## Significance statement

Social media platforms such as TikTok, Instagram, and YouTube have popularized a type of split-screen video known as “sludge content.” Sludge content consists of two videos stitched together to play simultaneously (i.e., split-screen). Typically, a primary educational or narrative video plays alongside a muted secondary video that is engaging but unrelated (e.g., footage of videogames or dominos falling over). Many viewers and commentators worry that this split-screen video format may harm attention and comprehension as a result of multitasking. Others have argued that the added stimulation could help sustain focus and benefit memory. Two controlled experiments were used to test whether the presence of a secondary video influenced learning from educational material. No evidence was found that sludge content reduced comprehension. Neither did the sludge content enhance memory for the main video. Rather, performance was approximately the same between the normal single video and split-screen “sludge” presentations. Furthermore, the sludge content did not consistently alter the viewers’ self-reported attention, interest, or perceived mental load. These results challenge the assumption that highly stimulating media necessarily harm learning and highlight the importance of empirically examining the influence of multimedia design features on cognition in contemporary digital environments.

## Split-screen distraction: the role of extraneous visual demands in learning from video

Split-screen videos are becoming a common element of popular social media platforms, such as TikTok, YouTube, and Instagram, which are heavily used by young adults (Bestvater, 2024). The particular style of split-screen videos common on these platforms juxtaposes a primary video, usually with someone speaking about a personal life experience or explaining something (e.g., makeup tutorials), while a muted repetitive secondary video plays simultaneously below. As noted by Fares (2025), this secondary video often uses gameplay footage from popular videogames (e.g., Subway Surfers, Minecraft) or other relaxing scenes (e.g., dominos falling, balls rolling down a ramp). Although the reasons for the algorithmically supported popularity of sludge content is somewhat murky, it is typically hypothesized that these videos are posted on social media platforms, because they increase viewer retention (Weaver, 2023).

This particular genre of split-screen video has received the pejorative, yet widespread designation of “TikTok sludge” or “sludge content.” The existence and popularity of these videos have received varied, yet often intense reactions from elements of the public. A recent *Scientific American* article, titled “Sludge Videos Are Taking Over TikTok—And People’s Mind,” puts forth a skeptical view of the content, suggesting that multitasking may hurt watchers’ attention spans in the long run—however, the article notes that research is needed on these popular social media trends, and that very little existed as of its publication in early 2024 (Mattson, 2024). Indeed, concerns about the negative effects of sludge content on attention is a common theme in most popular discussions of this trend (e.g., D’Anastasio, 2023; Lingenfelter, 2023). But this view is not universal. For example, well-known science communicator, Michael Stevens, has argued that split-screen video watching mirrors the types of activities that humans have long engaged in prior to social media, saying “we get panicky about attention spans … before TikTok, before the Internet—people would like talk to each other [and] watch the birds at the same time … people would divide their attention. They would talk on the phone, and also watch the cars passing by on their street. That’s sludge content pre-Internet” (Smosh Alike, 2023). Others have put forth an even more positive view of sludge content, arguing that the secondary videos might help to maintain learners’ calm, improve their attention, and ultimately benefit their memory for the content they are consuming (Fares, 2025; Pawlak, 2025). These conflicting views of sludge content and the associated concerns about the impact of this content on viewer’s learning and memory call for theoretically motivated, applied research into its effects.

## Split-screen videos, attention, and multimedia learning theory

Though popular opinions on the effects of sludge content on human cognition are far from consensus, psychological theory can also help anticipate the effects of split-screen video consumption on human memory and attention. Fundamentally, split-screen videos are a form of multimedia, usually combining one audio track and (at least) two independent sets of moving images, but sometimes more. As such, multimedia learning theory (Mayer, 2002, 2024) and perceptual load theory (Lavie et al., 2014) can be used to make informed theoretically backed predictions concerning the effects of this trend.

### Overload and underload hypotheses

As summarized by Sümer et al. (2025), there are two general hypotheses concerning the effects of consuming multiple forms of media simultaneously: the *overload* and *underload hypotheses*. The overload hypothesis states that having multiple videos playing at once will lead to decreased mnemonic encoding as a result of overloading the viewers’ attentions. Conversely, the underload hypothesis puts forth the notion that additional stimulation in the form of the secondary video might lead to increased focus, as the viewer might not seek other even more counterproductive forms of stimulation (e.g., mind-wandering, checking one’s phone).

As exemplified by Mattson (2024), the overload hypothesis is relatively intuitive. Indeed, generally multitasking impairs performance (May & Elder, 2018) and this phenomenon does not seem to get better with practice, as frequent media multitaskers are actually more susceptible to distraction (Ophir et al., 2009). However, findings in cognitive science surrounding selective attention and perceptual load theory suggest that the results of the split-screen video task might not be as negative as often assumed. Research on selective attention—the ability to focus one’s attention on a specific target stimulus even in the midst of other distractor stimuli—has shown that people are able to focus on relevant material and block out the influence of distracting stimuli (Egeth, 1967; Johnston & Dark, 1986; Makov et al., 2023), sometimes to a surprising extent (Simons, 2000; e.g., Simons & Chabris, 1999; Wood & Cowan, 1995). In the case of listening to two separate audio streams, one in each ear, long-standing results from these dichotic listening tasks indicate that listeners “experience[] no difficulty in listening to either speech at will and ‘rejecting’ the unwanted one” (Cherry, 1953, p. 977), offering hope for viewers of split-screen videos.

Perceptual load theory suggests processing will be impaired if the perceptual demands of the task are too high. Furthermore, the processing of distractor stimuli depends on the perceptual and cognitive demand of the task at hand (Murphy et al., 2016). In classic studies of perceptual load, participants are commonly asked to complete a focal task, while ignoring peripheral distractors, such as letters or shapes appearing elsewhere on the screen (e.g., Lavie, 1995). In these experiments, when task demand is high and the distractor stimuli is irrelevant to the focal task, then attentional selectivity will tend to occur relatively early, meaning very little attention will be paid to the distractor (in this case the secondary video). When cognitive and perceptual load of the primary task is low, such as perhaps in the case of listening to a speaker describe mundane events, more attention might be given to the distractor stimulus. In other words, perhaps attentional resources can adapt to the demands of the task, and one should not expect large effects of a distractor should this adaptive capability not be exceeded.

There is also empirical evidence for the underload hypothesis. Here the evidence comes from research on task vigilance and mind-wandering (Sümer et al., 2025), with the primary finding that vigilance can decrease as the time spent on the same task increases (See et al., 1995). This theory applied to the sludge content being that in the absence of the sludge viewers would become habituated to the primary video and thus their attention would wander. When the secondary distractor video is added, this might prevent the viewer’s attention from wandering and thus actually increase performance (Fares, 2025). This hypothesis might also be interpreted in light of research finding that brief mental breaks through momentary task switching can increase task vigilance (Ariga & Lleras, 2011). In this way, one could view the ability to momentarily watch the secondary distractor video as a means of offering the viewer a brief break through which slips in task vigilance could be remediated.

### Split-screen videos and the split attention effect

These attentional dynamics also intersect with work from multimedia learning theory, particularly the *split attention effect* (Mayer & Moreno, 1998; Schroeder & Cenkci, 2018), which states that multimedia learning environments should not require learners to integrate information from multiple sources concurrently. For example, Ayres and Sweller (2005) describe the difficulties learners will have if they need to integrate between the text of a geometry problem and the problem solution’s solution if both sources of information are spatially separated. It is thought that this separation increases extraneous cognitive load, and thus potentially overwhelms the learners’ working memory. This source of cognitive load can often be circumvented in video environments by replacing on-screen text with narration (Mayer & Moreno, 1998). Indeed, multimedia environments should be designed such that information is conveyed in an integrated fashion.

Split-screen videos present an interesting case, because they seem at first glance to controvert multimedia principles; the genre leans into extreme stimulation in a way that seems like it would assuredly lead to split attention and thus decreased ability to encode the to-be-learned information. However, upon closer examination the format does seem to follow some of the multimedia principles, even if by trial and error. Most saliently, sludge content typically contains a single audio source, as one video is typically muted. Furthermore, the primary video is usually visually uninformative, consisting of a narrator speaking to the camera. In this sense, even though there are two videos playing, there is only one important audio channel and one important (or at least stimulating) visual channel.

It should also be acknowledged that one of the main tenets of the split attention effect is that learning is impaired due to the need to *integrate* across spatially or temporally separate material (Ayres & Sweller, 2021); however, in the sludge content, there is usually no need to integrate information from the secondary video with that of the first. Ostensibly, the secondary video is included simply to provide an additional source of visual stimulation, with low cognitive demands (Weaver, 2023). Another interesting aspect of the sludge genre is that the secondary distractor video is usually non-realistic footage from videogames, perhaps reflecting the fact that under some conditions realistic imagery has been found to impair retention independent of split attention effects (Skulmowski, 2023b).

Although there is little research on social media-style split-screen videos from the perspective of human memory (though see Pawlak, 2025 for a small-scale *n* = 6 EEG study), Fares (2025) has recently reported results of a within-participants (*n* = 24) study of sludge content on comprehension of a personal narratives paired with gameplay footage from the videogame Minecraft. This study overlaid audio narratives and subtitles over a single type of distractor (Minecraft gameplay), comparing it to audio only and subtitles only conditions. As such, it was not testing the true split-screen video content, typical of the sludge content discussed in the present study, as there was only ever one video playing. The main effect of the distractor video was small (*η*^2^_*p*_ = 0.002) and nonsignificant. The use of a single type of distractor also raises the question of whether the (null) effects were limited to the stimulus used (Yarkoni, 2022).

Interestingly, the null results of Fares (2025) are not uncommon in the general research domain of simultaneous multimedia stimulation. This result mirrors other research which failed to find a significant impact of subtitles on learning from video (Cojean & Martin, 2021). This also matches findings by Sümer et al. (2025) who found that listening to background music while studying did not significantly affect task performance or attention.

Given the lack of well-powered studies on sludge content, the popular interest in this phenomenon, the disparate predictions it elicits with regard to multimedia comprehension and learning, it seems as though a controlled study of this contemporary form of multimedia could provide nuanced evidence to the discussion of so-called sludge content.

## Present study

In the present series of within-participants studies, the effects of split-screen videos on comprehension and memory for short (7–15 min) video lectures are tested. In Study 1, TED talks are used as the primary stimuli of interest. Though these videos have imagery associated with them, it tends to be minimalistic and on-screen for relatively long periods of time. In Study 2, more visually taxing animated content aimed at conveying scientific information is used as stimuli. Here, important information is conveyed both via the animation and the accompanying voiceover. In Study 2, we also include secondary distractor videos that present their own unrelated text to further manipulate the potential effects of visual load.

The potential implications of this series of studies are expanded upon by (a) capturing self-reported measures of engagement and mental demand, and (b) checking for moderation by age of participant, as short-form video content is typically associated with young adult viewers, ages 18–34 (Bestvater, 2024). Together, these studies allow us to examine whether the impact of split-screen distractions depends on the characteristics of the viewer or underlying visual demands of the lecture material, thereby providing insight into the conditions under which such media may hinder or leave unaffected comprehension and memory. Theoretically these studies contribute to the development of multimedia learning theory, while practically helping parents, educators, and social media consumers better understand the effects of the split-screen content that is being increasingly consumed across video-based social media.

## Study 1: video lectures with slides

### Data and materials availability

Both Studies 1 and 2 were preregistered. Preregistrations, open data, and analysis files can be found on the Open Science Framework, https://osf.io/adbkc/.

### Methods

#### Participants

A simulation-based power analysis, based on sampling new data and refitting the focal logistic hierarchical linear model of interest, indicated that a sample size of 75 participants would be sufficient to detect a small effect *d* = 0.20 with more than 85% power. Thus, the target *N* = 75 was preregistered. As specified in our preregistration, our sample came from two sources: an educational psychology subject pool at a large university in the American Mountain West and Prolific, and an online participant pool where experiments can be completed in exchange for compensation. Prolific participants were recruited from English-speaking countries (Australia, Ireland, USA, and UK).^1^ It should be noted that because this experiment was conducted within subjects, the usage of two participant pools would not contribute unmeasured confounds to the study relevant to estimating the main effect of the split-screen videos; however, it could lead to confounds related to the age analyses. This issue is resolved in Study 2. All data, regardless of source, were collected online, from a location of the participants’ convenience.

Participants (*N* = 75) were recruited from two sources: the university subject pool (*n* = 42) and Prolific (*n* = 33). Ages ranged from 18 to 65 years (*M*_Age_ = 31.1, Median_Age_ = 23, SD = 14.5). Of those who provided demographic information, 44 identified as female, 27 as male, and 3 as non-binary. Participants identified primarily as white (55%), followed by Black (7%), Hispanic or Latinx (6%), and Asian (5%).

#### Stimuli

Two videos from the TED lecture series were selected as the primary stimuli of interest. One video presented by Rajesh Rao (Rao, 2011) discussed computational linguistics, and the other video by Kevin Slavin (Slavin, 2012) centered around the impact of algorithms on the world. These videos had been previously used in video learning studies by Mueller and Oppenheimer (2014). As common in TED lectures, the videos depict a single lecturer presenting a polished lecture on their topic of expertise between 15 and 20 min in length. The videos toggle between shots of the lecturer, supplemented by close-ups on the presentation slides. These videos were selected as self-contained stand-ins for the type of content that might be presented in a college class.

Two distractor videos were used, but each participant only saw one of these distractors. One distractor featured gameplay from the videogame Subway Surfers, depicting a repetitive gameplay loop of a character jumping from railcar to railcar while avoiding obstacles and collecting coins. The other distractor video showed footage from the videogame Minecraft. This video featured a character navigating a long obstacle course sequence composed of floating blocks and different biomes. These distractor videos were selected, because Minecraft and Subway Surfers are commonly cited as prototypical examples of the type of videos used in TikTok sludge content (e.g., D’Anastasio, 2023; Mattson, 2024). The order of the videos, the presence of the distractor video, and the chosen distractor video were randomly manipulated between participants.

#### Procedure

**Fig. 1:**
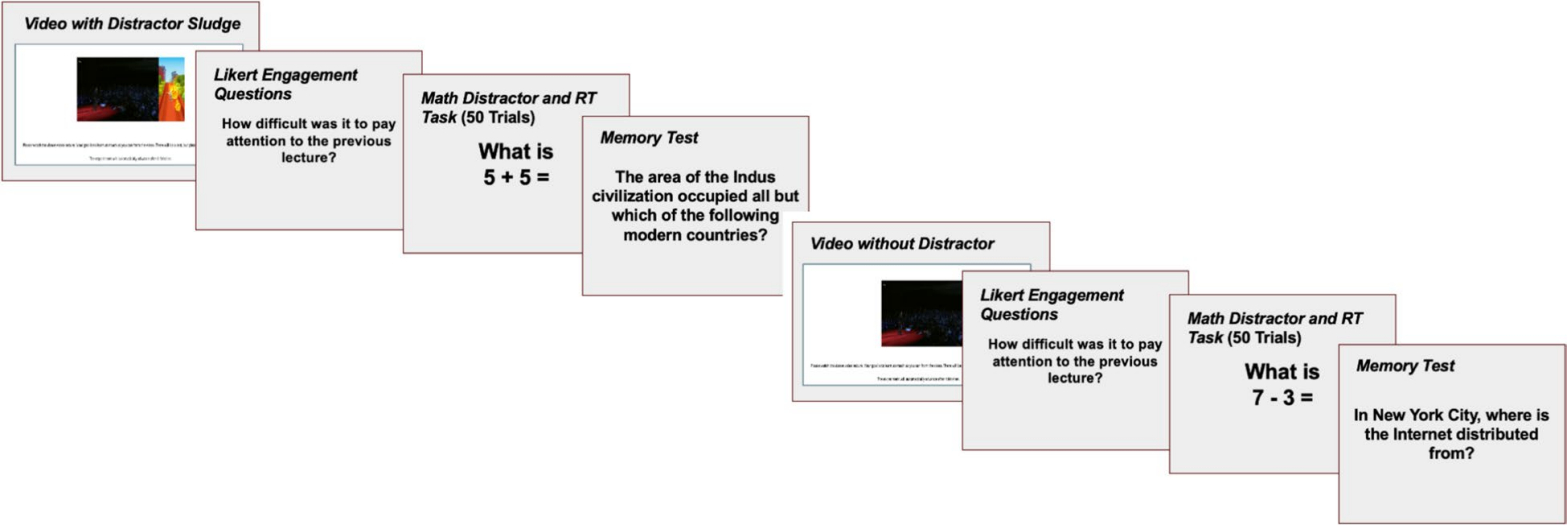
General Procedure Timeline for Studies 1 and 2. *Note* Each participant completed both the split-screen and traditional conditions. The order of these conditions, as well as the content of the primary (lecture) and secondary (distractor) videos, were randomly assigned between participants, with the constraint that no duplicate videos were presented within the tasks completed by a given participant. The memory test for each video consisted of 10 multiple-choice questions

The first experiment followed a 2 × 2 × 2 × 2 mixed design comprising one within-subjects factor (video type: traditional vs. split-screen) and three between-subjects methodological factors related to stimulus assignment. These factors were presentation order (traditional first vs. split-screen first), assignment of lecture stimuli to condition, and choice of distractor stimulus (Minecraft vs. Subway Surfers). Figure 1 summarizes the procedure used in Studies 1 and 2.

As shown in Fig. 1, the primary split-screen versus traditional video manipulation was performed within subjects. Each participant watched two videos in a random order. One of these videos was randomly assigned to have a distractor video playing simultaneously during the entire runtime (upon ending distractor videos looped to the beginning). When the distractors were shown, participants were told to focus on learning the content of the video lecture, which always appeared on the left-hand side of the screen. In the traditional non-distractor condition, the video lecture was shown in the middle of the screen.

After watching each video, the participant answered two engagement questions assessing interest and attentional difficulty. Following each learning phase, participants completed a short distractor task, which was included to disrupt rehearsal and prevent active maintenance of the learned material in working memory. These distractor activities were composed of 25 simple one-and-two digit math problems (e.g., “What is 7–4?”) randomly interleaved with 25 reaction time questions. During the math questions, the participant was given a textbox to indicate the correct answer (e.g., by typing 3 and hitting enter). During the reaction time questions, participants saw four squares arranged horizontally. One of these squares was always assigned a slightly darker color than the rest. The participant was required to answer using the number keys 1, 2, 3, or, 4 to indicate from left to right which square was filled with a different color. On average these 50 distractor trials were completed in approximately a minute and a half after the first video (*M*_Time_ = 96 s, Median_time_ = 94 s, SD = 20 s). Participants finished these 50 trials slightly faster after the second video (*M*_Time_ = 88 s, Median_time_ = 85 s, SD = 18 s).

After finishing the 50 total distractor trials, the participants were presented with a multiple-choice and true/false comprehension test. Each video was associated with 10 or 11 comprehension questions. These questions were developed by the authors and designed to test the participants’ memory for facts stated directly in the respective video lecture. For example, one test question for Slavin’s (2012) talk asked “What do Epagogix’s algorithms claim to be able to do?” With the correct answer being: “Predict box office success.” Full test banks can be found on the Open Science Framework repository associated with this paper.

After completing the multiple-choice test that matched the video they had just seen, they watched the second video. For each participant, the condition of the first video was randomly assigned, and the second video was assigned to the complementary condition, ensuring that each participant experienced both conditions exactly once. After the second video, the same blocks of trials were completed: engagement questions, distractor task, and comprehension test for the second video. After completing the second comprehension test, the participants filled out a short survey. The final survey captured demographic information and asked whether there were any difficulties with following task instructions or other technical issues that arose. All participants gave informed consent, and ethical approval was given for the present series of studies by the author's Institutional Review Board (#IRB_00181555).

### Results

The following analyses were preregistered. All analyses were performed at the item level in R using the *lme4* package for multilevel modeling. All modes included random intercepts for participant and question ID. Specifically, for the focal test comprehension models, responses were nested within participants and test items. Thus, models included random intercepts for both participants and items. This approach accounts for the non-independent data structure, while accommodating an unequal number of items across videos.

#### Test performance

Overall test performance was relatively evenly distributed between 0 and 100%, *M* = 0.62, *Median* = 0.70, SD = 0.24. Reliability for the test for the Slavin video was *α* = 0.61 and for the Rao video was *α* = 0.76. Mean performance in the split-screen condition (*M* = 0.61, *Median* = 0.64, SD = 0.24) was slightly lower than the traditional condition (*M* = 0.63, *Median* = 0.70, SD = 0.24, *d* = − 0.08). A mixed-effects logistic regression was used to test whether accuracy differed by video type (traditional vs. split-screen). The effect of adding the split-screen video was not statistically significant, *log *OR = 0.17, SE = 0.12, *z* = 1.43, *p* = 0.15, indicating that accuracy did not differ across conditions. The corresponding average marginal effect, indicating an average difference of 3% (AME = 0.03, 95% CI = [− 1%, 7%], *p* = 0.15), was small and nonsignificant. Follow-up robustness checks did not find a significant interaction between order of conditions (i.e., traditional first vs. split-screen first) and condition, χ^2^(2) = 1.96, *p* = 0.38.

A follow-up model tested whether the effect of video type was moderated by participant age. The model revealed a significant age-by-video-type interaction, *log *OR = 0.02, SE = 0.01, *z* = 2.05, *p* = 0.04. Inspection of the coefficients suggested that performance on traditional videos tended to increase with age, whereas performance for split-screen videos decreased with age. Neither the main effect of age (*log *OR = − 0.01, SE = 0.01, *z* = − 0.52, *p* = 0.60) nor the main effect of video type (*log *OR = − 0.51, SE = 0.36, z = − 1.44, *p* = 0.15) was significant. In line with recommendations by von Hippel and Schuetze (2025), Study 2 attempted to replicate this interaction as further described below; however, this replication attempt was not successful.

#### Self-report measures of engagement

Self-report data were analyzed using mixed-effects linear models with random intercepts for participant and video. Participants reported slightly greater difficulty maintaining attention during the split-screen videos than during the traditional videos, but this effect was not statistically significant, *b* = − 0.29, SE = 0.17 *t*(73) = − 1.68, *p* = 0.10. Likewise, self-reported interest did not differ significantly by condition, *b* = 0.24, *t*(74) = 1.56, *p* = 0.12, though traditional videos were rated as directionally more interesting.

## Study 2: increased visual load of primary and secondary videos

Because Study 1 yielded no main significant effects of the sludge content on comprehension, in Study 2 it was tested whether increased visual load stemming from more complex stimuli would potentially elicit split attention costs.

### Methods

#### Participants

Given the null effects, found in the previous study, sample size was increased for the second study. In line with our preregistered recruitment target, participants (*N* = 100) were recruited from the online participant platform Prolific. Based on the same simulation as used to assess power in Study 1, a post hoc power analysis indicated that a study with this sample size should detect an effect of *d* = 0.20 with more than 90% power. This power analysis was not used to determine the sample size and is reported for informational purposes only. Participants were recruited from English-speaking countries (Australia, Ireland, the USA, and the UK). On average, participants were middle-aged (*M*_Age_ = 42.1; Median_Age_ = 40.0, SD = 13.66). Most participants were women (*n* = 54). The majority of participants were white (*n* = 68), followed by Black (*n* = 19), Asian (*n* = 6), Hispanic and or Latinx (*n* = 3), Native Hawaiian and/or Pacific Islander (*n* = 1).

#### Stimuli

The primary difference between Studies 1 and 2 was related to stimuli selection. In Study 2, four primary video lectures were used. Unlike Study 1, these video lectures were not filmed in a TED-talk-style format, but rather made extensive use of animated sequences and much more minimal (or sometimes no) depiction of the narrator. Like the TED talks, these videos exhibit high production value. The new stimuli were also selected to be more recently created, having been produced between 2014 and 2025. Videos were gathered from popular science communication YouTube channels, with high production values (CrashCourse, Kurtzgesagt, and StudyHall). Full listing of the videos used as stimuli can be found on the OSF project associated with this paper. Video topics were drawn from STEM and social science topics (e.g., the function of bacteriophages, and visual perception). These videos are typically designed to supplement high school and college courses; for example, the StudyHall video concerned Piaget’s stages of cognitive development and was produced in partnership with Arizona State University.

In Study 2, the pool of possible distractor videos was increased from two to eight. Four were relatively typical distractor videos for sludge content, consisting of gameplay footage, repetitive sequences of marbles falling, or depictions of animated fruit dancing (mirroring Study 1). The other four videos were lyric videos, a subgenre of music videos with animated depictions of the lyrics to a song. These lyric videos were included in our distractor bank in order to test whether distractor videos with more textual content would create perceptual overload leading to impaired performance. Each participant only saw one of these eight randomly sampled secondary distractor videos alongside one of the primary instructional videos.

#### Procedure

The procedure mirrored that of Study 1, except for two changes. First, the stimuli and associated comprehension test questions were changed as described in the Stimuli section above. As a result of these stimulus sampling changes, the second study followed a mixed design with one within-subjects factor (video type: traditional vs. split-screen) and one between-subjects factor (presentation order). To aid in generalizability of results (Yarkoni, 2022), stimuli also varied across participants: Lecture videos and distractor videos were randomly sampled for each participant from a pool of four lectures and eight distractors, respectively. The sampling was constrained such that no lecture was shown twice to the same participant. Second, an additional post-video measure of cognitive load was added, specifically the NASA task load index (NASA TLX)—mental demand question (Hart & Staveland, 1988). All other features of the procedure stayed the same.

### Results

As with Study 1, the following analyses were preregistered and performed using multilevel logistic and linear models in R using the package *lme4*.

#### Test performance

Overall test performance was slightly higher than that of Study 1, *M* = 0.69, *Median* = 0.70, SD = 0.21. In contrast to Study 1, mean performance in the split-screen condition (*M* = 0.70, *Median* = 0.75, SD = 0.22) was slightly higher than the traditional condition (*M* = 0.68, *Median* = 0.67, SD = 0.21, *d* = 0.09). Reliability for each of the four knowledge tests ranged between α = 0.63 and 0.67.

A mixed-effects logistic regression was conducted to examine the effect of the within-subjects video-type manipulation (traditional vs. split-screen distractor) on participants’ accuracy across questions, with random intercepts for participant and question. No significant effect of video-type manipulation was found, *log *OR = − 0.06, SE = 0.11, *z* = − 0.54, *p* = 0.59. The average marginal effect of video type was negligible (AME = − 0.01, 95% CI = [− 5%, 3%], *p* = 0.59), indicating that the average proportion correct differed by only one percent between conditions.

A follow-up robustness check was conducted to determine whether there was an interaction between order of the conditions and the conditions themselves. The omnibus likelihood ratio test comparing models with and without order was significant, χ^2^(2) = 8.16, *p* = 0.017. However, closer inspection revealed that neither the main effect of order nor its interaction with video type was statistically significant in the model including these terms (*p*s > 0.06). The interaction suggested that performance in the traditional condition was higher when it was presented first, *b* = 0.42, SE = 0.22, z = 1.86, *p* = 0.06, though this effect did not reach conventional levels of statistical significance. We note that no evidence for an order effect was found in Experiment 1.

#### Distractor type

An exploratory follow-up model further subdivided the conditions, analyzing whether the gameplay and repetitive (text-free) distractor videos differed from the text-heavy (lyric) videos in terms of comprehension test performance. These gameplay-style distractors were used in Experiment 1; however, the lyric video-style distractors were new to Experiment 2. Thus, it was of interest to determine whether the distractor type influenced participants’ test performance.

These analyses between the three dummy-coded conditions (traditional, split-screen gameplay, and split-screen lyrics) likewise showed no significant differences, as indicated by a likelihood ratio test, Δχ^2^(2) = 3.23, *p* = 0.20. Participants viewing the lyric videos condition showed descriptively higher accuracy compared to the videos without the distractors (AME = 0.06, 95% CI = [− 0.01, 0.13], *p* = 0.08). Participants viewing the gameplay footage distractors also showed descriptively higher accuracy compared to the videos without the distractors (AME = 0.02, 95% CI = [− 0.03, 0.08], *p* = 0.40). It should be noted that statistical power is relatively lower to detect effects of distractor type, due to the between-subjects nature of the present analysis.

#### Moderation by age

**Fig. 2:**
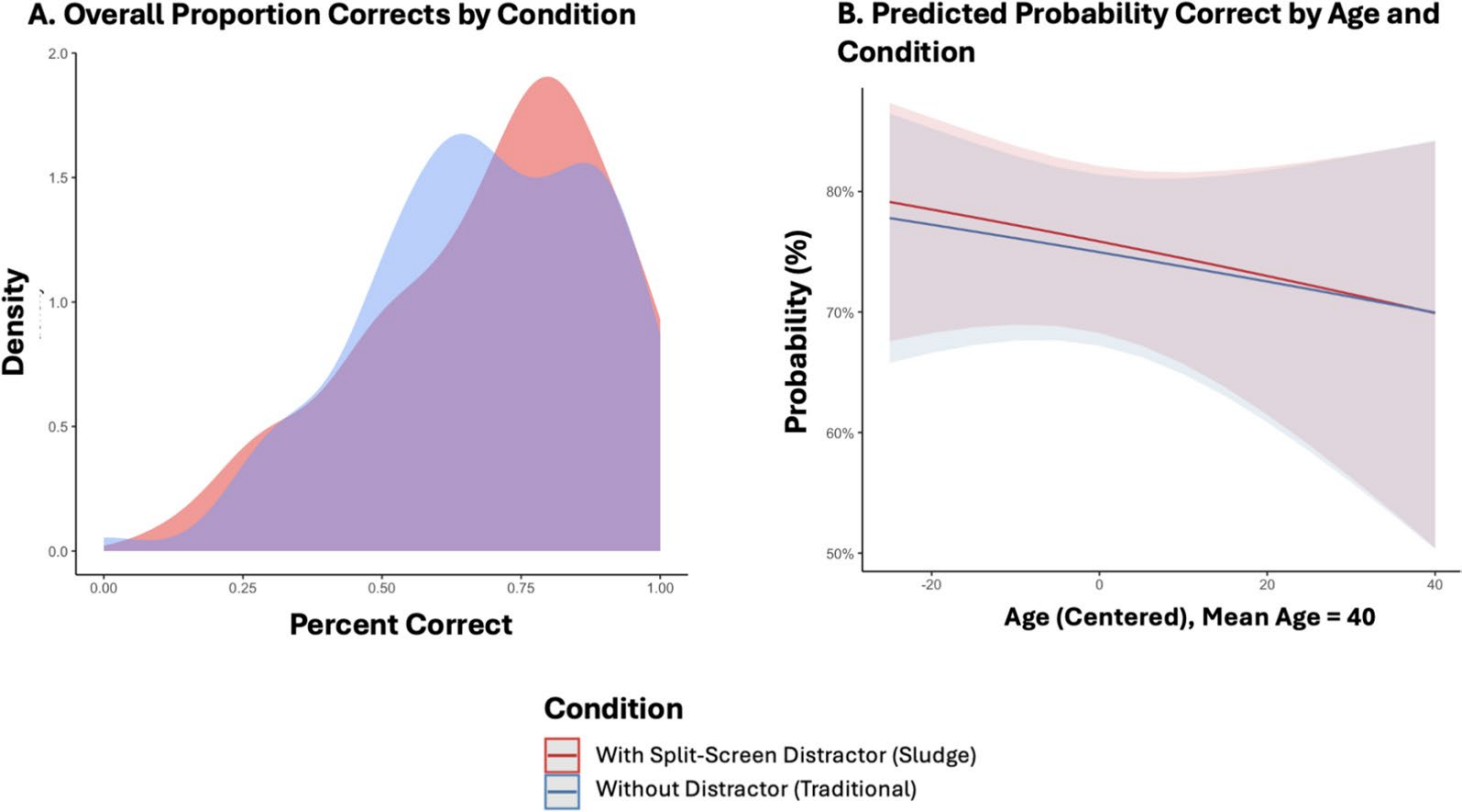
Results from Study 2. *Note* Panel A shows the overall distribution of proportion correct after averaging within participants by condition. Panel B shows the interaction plot between age and condition

To test whether age moderated effects of video type, a model including the interaction between centered age and video type was fit. Neither the main effect of age (*log *OR = − 0.01, SE = 0.01, z = − 0.80, *p* = 0.43) nor the interaction (*log *OR < 0.01, SE = 0.01, z = 0.15, *p* = 0.88) was significant, suggesting that age was not significantly associated with comprehension accuracy. The predicted probabilities from this model are shown in Fig. 2. In other words, the significant interaction from Study 1 did not replicate in Study 2.

### Self-reported measures

#### Attention difficulty

The mean attention difficulty in the split-screen condition was 2.51, while the mean in the traditional condition was 2.32 out of 5. Self-reported attention difficulty did not differ significantly between video types, *b* = − 0.19, *t*(99) = − 1.30, *p* = 0.20.

#### Interest level

Participants reported significantly higher interest for the traditional videos (*M* = 4.25) than for the videos presented split-screen (*M* = 4.02), *b* = 0.24, *t*(98) = 2.18, *p* = 0.03. This difference remained significant even when accounting for a potential interaction with age, *b* = 0.29, *t*(88) = 2.54, *p* = 0.01; however, no significant main effect of age nor interaction between age and condition was found (*p*s > 0.05).

#### Perceived task load

Mean task load, as operationalized by the NASA TLX, was slightly higher in the traditional condition (*M* = 2.26), compared to the split-screen condition (*M* = 2.13). However, task load did not significantly differ by video type, *b* = 0.13, *t*(97) = 1.26, *p* = 0.21. A weak interaction with age—if interpreted—would suggest a potential trend toward increased task load with increasing age for the split-screen video condition, *b* = − 0.01, *p* = 0.09.

## Discussion

The rise of split-screen “TikTok sludge” or “slop” videos has led to questions in the popular media concerning the effects of these forms of multimedia on learning and comprehension. Some commentators have argued that these videos will lead to reduced memory performance due to perceptual overload, while others have argued that these videos will have no effect (Smosh Alike, 2023) or actually improve focus by keeping the viewer stimulated (Fares, 2025; Pawlak, 2025). Indeed, these split-screen videos present an interesting task to study from the lens of multimedia learning theory given their potentially intense demands on the viewer’s attention.

Yet in the present study, no statistically significant effects of these types of videos on learning was found. In Study 1, the average marginal effect of the distractor video on the comprehension test was negative 3% (95% CI = [− 1%, 7%]), while in Study 2 the average marginal effect was positive 1% (95% CI = [− 5%, 3%]). While the present analyses cannot rule out *an* effect of split-screen videos, these confidence intervals are not consistent with a large effect of these videos (i.e., > +/− 10% on comprehension tests in the short-term), as long as multimedia presentation follows the general form tested in the present study. Critical to note is the feature of sludge content where the secondary video is muted and contains no relevant information to be later tested. Thus, viewers do not need to integrate information from across the two videos. Another potential important feature is that the secondary video does not usually contain images of people or faces, which have been found to disrupt selective attention (Murphy et al., 2016).

To summarize, our findings suggest that the presence of a secondary, visually engaging but task-irrelevant video did not meaningfully impair or enhance comprehension, attention, or perceived cognitive load. One possible interpretation is that this pattern is consistent with perspectives from cognitive load and multimedia learning theory, which place emphasis on the integration of information from multiple sources as a source of load (Ayres & Sweller, 2005; Mayer, 2002; Verhoeven et al., 2009). In the present task, learners were not required to integrate information from the distractor stimuli with the instructional content, which may explain why the distractor had little effect on comprehension or self-reported cognitive load. However, future analyses using eye-tracking or online attentional measures could result in improved understanding of potential cognitive mechanisms implicated in the present task.

With respect to the cognitive load as an outcome measure, it is important to note that self-report measures constitute only one, albeit widely used, type of assessment and may not accurately reflect more subtle or transient load-related processes (Schuessler et al., 2026). Furthermore, even different self-report measures of cognitive load may not always agree with one another (Skulmowski, 2023a). As opposed to the one-item NASA TLX used in the present study, it could be preferable to use more in-depth measures of cognitive load in future research (e.g., Leppink et al., 2013). Thus, the present findings do not rule out the possibility that future work using more sensitive self-report or neuroscientific measures could identify cognitive load-related effects not observed here (Murphy et al., 2016).

Although the primary comparison of interest—memory test performance across conditions—showed nonsignificant results, two weak, and somewhat idiosyncratic effects were found to be statistically significant in at least one of the two present studies. Specifically, in Study 1, a small interaction between video type and age was found, suggesting that older participants learned less from the sludge videos. However, this did not replicate in Study 2. Although one cannot exclude the possibility of potential age-related moderation in a larger sample or with a different population, the present studies do not strongly support hypothesized moderation effects by age. Similarly, in Study 2 small negative effects of the split-screen videos on interest were found; however, this effect was not present in Study 1. However, it should be noted that both these statistically significant results would not survive corrections for multiple comparisons. Thus, the present series of studies did not find strong or replicable effects of split-screen videos on any of the present behavioral or self-report outcomes of interest (see von Hippel & Schuetze, 2025).

Despite their apparent violation of multimedia design principles, split-screen “sludge” videos may not fundamentally disrupt learning when the primary information source is clear and auditory attention is allowed to focus on a single source. These results are in line with recent findings about background music during learning (Sümer et al., 2025). Furthermore, the findings highlight the resilience of cognitive processing under complex visual stimuli. More directly, the present studies suggest that concerns about the immediate cognitive costs of “sludge” content, as expressed by popular commentators (e.g., D’Anastasio, 2023; Mattson, 2024), on memory may risk being overstated. Future research should examine whether these null effects persist under more demanding learning materials, varying lengths of exposure, longer retention intervals, or when the secondary video competes for auditory as well as visual attention.

## Data Availability

Preregistrations, open data, and analysis files can be found on the Open Science Framework, https://osf.io/adbkc/.

## Footnotes

1 As a robustness check to determine if sample source affected the present results, a model including the main effect of sample source (Prolific versus university sample) and its interaction with condition was tested. This model did not significantly improve fit over the nullmodel without sample source, χ^2^(2) = 4.75, p =.093, suggesting no significant moderation of the effect of video type by recruitment source. Accordingly, the present analyses report results collapsed across source.
